# Membrane vesicle production via cell-to-cell communication-induced autolysis in *Streptococcus mutans*

**DOI:** 10.1128/spectrum.00334-25

**Published:** 2025-05-23

**Authors:** Ryo Nagasawa, Tamami Ito, Chika Yamamoto, Mio Unoki, Nozomu Obana, Nobuhiko Nomura, Masanori Toyofuku

**Affiliations:** 1 Graduate School of Life and Environmental Sciences, University of Tsukuba 98393 , Tsukuba, Ibaraki, Japan; 2 Graduate School of Science and Technology, University of Tsukuba 624301 , Tsukuba, Ibaraki, Japan; 3 Faculty of Medicine, Transborder Medical Research Center, University of Tsukuba 622602, Tsukuba, Ibaraki, Japan; 4 Microbiology Research Center for Sustainability, University of Tsukuba 623473, Tsukuba, Ibaraki, Japan; 5 Faculty of Life and Environmental Sciences, University of Tsukuba 13121, Tsukuba, Ibaraki, Japan; 6 Tsukuba Institute for Advanced Research, University of Tsukuba, Tsukuba, Ibaraki, Japan; Cinvestav-IPN, Mexico City, Mexico

**Keywords:** membrane vesicle, cell-cell communication, autolysis, *Streptococcus mutans*

## Abstract

Membrane vesicles (MVs) released from bacteria into the extracellular environment play various roles in bacterial and bacterial–host interactions. In Gram-positive bacteria, cell wall degradation or damage is crucial for cytoplasmic MV (CMV) release because the cytoplasmic membrane must pass through the thick cell wall to the extracellular space. Although *Streptococcus mutans*, a major causative agent of dental caries, is known to release CMVs that are involved in pathogenicity and biofilm formation, the mechanism by which CMV production is triggered is unknown. In this study, we demonstrate that *S. mutans* induces CMV release via the Com system, a cell-to-cell communication system mediated by peptide signals. CMV release is primarily dependent on autolysin encoded by *lytF*, which is regulated by the Com system. Electron microscopy observations indicated that CMV release was accompanied by cell death in a subpopulation of cells. We compared the protein profiles of CMVs, showing that CMVs induced via the Com system contained not only secreted proteins but also cytoplasmic proteins specifically expressed during Com system activation. We further demonstrate that the functionality of CMVs in biofilm formation differed depending on how they were induced.

**IMPORTANCE:**

Bacteria release membrane vesicles (MVs) that are involved in diverse biological processes such as cell-to-cell communication and also affect the bacterial host through their immunomodulatory activity. Recent studies have focused on elucidating the mechanisms underlying MV formation. In Gram-positive bacteria, it has been shown that cell death represents a major pathway for MV formation. Since cell death would not benefit the dying cell, but may provide benefit to the remaining cells, it is essential to understand the regulatory mechanisms governing the formation of MVs at the population level. Here, we show that MV formation in *Streptococcus mutans* is regulated by cell-to-cell communication. A subpopulation of cells triggers cell death-meditated MV formation, from which the remaining cells derive benefits. This is the first report showing cell-to-cell communication regulates MV formation in Gram-positive bacteria that would provide insights into the regulatory mechanisms governing cell death-mediated MV formation at the population level.

## OBSERVATION

**Fig 1:**
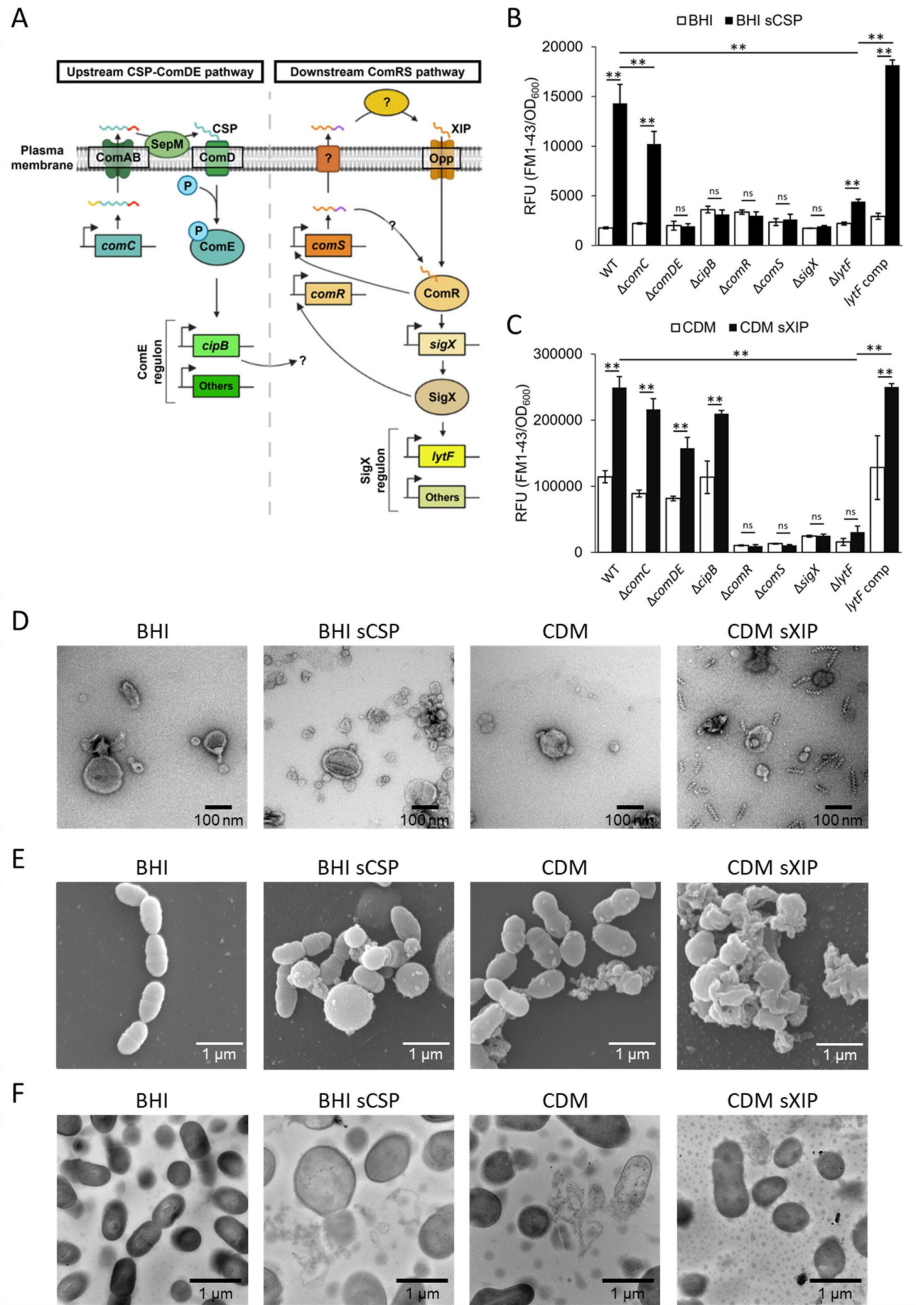
Quantification and observation of CMVs induced by the Com system. (**A**) The Com system of *S. mutans* consists of the CSP-ComDE pathway and the ComRS pathway (8–10). The CSP precursor encoded by *comC* is processed by the ComAB transporter and the extracellular protease SepM, resulting in mature CSP. Mature CSP is recognized by the two-component system ComDE, which induces expression of the ComE regulon including *cipB. cipB* is essential for the connection from the upstream CSP-ComDE pathway to the downstream ComRS pathway, although the mechanism is unknown. The XIP precursor is encoded by *comS*. XIP precursor is secreted into the extracellular environment by unknown mechanisms and then processed. Mature XIP is taken up intracellularly by oligopeptide permease. There appears to be a pathway by which XIP precursor matures without extracellular secretion. Mature XIP binds to ComR and induces expression of *sigX*, which encodes a sigma factor. SigX regulons include the autolysin gene (*lytF*), late competence genes, and other cellular function genes. ComR and SigX upregulate *comS* and *comR* expression, respectively. (**B and C**) Cells were grown in an aerobic atmosphere containing 5% CO_2_ at 37°C for 24 h. sCSP and sXIP were added at final concentrations of 1 µM and 10 nM, respectively. The collected CMVs were labeled with FM1-43FX and quantified. The fluorescence values were normalized by the OD_600_ of 24 h cultures. These data represent the means ± SD of the results from three independent experiments. The asterisks indicate a significant difference (**: adjusted-*p* <0.01; Tukey’s HSD test, ns: not significant). (**D**) CMVs purified from 24 h cultures by density gradient centrifugation were observed by TEM. Scale bars indicate 100 nm. (**E and F**) Cells were grown in an aerobic atmosphere containing 5% CO_2_ at 37°C for 24 h. sCSP and sXIP were added at final concentrations of 1 µM and 10 nM, respectively. Cells in each culture condition were observed by SEM (**E**). Ultrathin sections of cells were observed by TEM (**F**).

Bacterial extracellular membrane vesicles (MVs), which mainly contain cell-derived components, serve as a communication tool between bacteria or with host cells (1). In Gram-positive bacteria, cell wall integrity is an important determinant for cytoplasmic MV (CMV) release (2, 3). *Streptococcus mutans*, a major caries-causing Gram-positive bacterium, also releases CMVs that have been suggested to have pathogenic functions (4). However, the mechanisms underlying the release of CMV remain unclear. Since *S. mutans* has a robust cell wall, cell wall degradation could be a key step in CMV release; however, phage-derived endolysin, which has been reported to cause CMV formation by bubbling cell death (2, 5), is not encoded in the *S. mutans* UA159 genome. A recent study showed the involvement of autolysins in CMV formation in *B. subtilis* (6). *S. mutans* induces autolysin expression via the Com system, a cell-to-cell communication system mediated by peptide signals: competence-stimulating peptide (CSP) and SigX-inducing peptide (XIP) (Fig. 1A) (7). The Com system is a hierarchical gene expression pathway with an upstream CSP-ComDE pathway and a downstream ComRS pathway that regulates genetic competence, autolysis, and other functions (8), but its involvement in CMV formation has not been studied to date.

In this study, we demonstrated that the Com system leads to the release of CMVs containing cytoplasmic proteins via LytF-dependent lysis of a subpopulation of cells. Our results reveal a mechanism for CMV release in *S. mutans* and provide new insights into how cell death- meditated MV formation is regulated at a population level.

In this study, brain heart infusion (BHI) and chemically defined medium (CDM) were used as complex and synthetic media, respectively, because the response of *S. mutans* to peptide signals is limited to either CSP or XIP, depending on the medium (8, 9). The Com systems were induced by the addition of synthetic CSP (sCSP) and XIP (sXIP). The exogenous addition of these signals significantly increased CMV formation in UA159 wild-type (WT) (Fig. 1B and C). Transmission electron microscopy (TEM) results showed typical CMV structures (Fig. 1D). The spirosome-like structures reported in *Escherichia coli* have also been observed in CDM sXIP (11). Cell-attached CMVs were observed by scanning electron microcsopy (SEM) under all conditions except for BHI, where CSP was not added (Fig. 1E). The large spherical structures reported by Dufour et al. and Qi et al. (12, 13) were also observed, especially when sCSP was added (Fig. 1E). In addition, cell debris, indicative of cell death, was observed in conditions except BHI where sCSP was not added (Fig. 1E). TEM analysis of ultrathin sections further suggested that CMV release was accompanied by cell death that occurred in a subpopulation of cells (Fig. 1F).

CSP and XIP induce the expression of more than 80 genes (8). Therefore, we attempted to identify the genes required for CMV release using deletion mutant strains. To examine the effect of native CSP, the CMV production of Δ*comC* lacking the CSP precursor was compared with that of the WT (Fig. 1B and C). The Com system is merely induced in BHI medium (7), and the deletion of *comC* had no effect on CMV production (Fig. 1B). The WT produced more CMVs than Δ*comC* when sCSP was added (Fig. 1B), presumably due to an indirect induction of CSP production in the WT. CSP is recognized by the two-component system ComDE, and signaling is transmitted to the downstream XIP pathway via *cipB* (14). In BHI, Δ*comDE* and Δ*cipB* did not produce CMVs in response to sCSP, whereas in CDM, they were produced in response to sXIP (Fig. 1B and C). The XIP pathway-deficient strains Δ*comR*, Δ*comS*, and Δ*sigX* did not produce CMVs in response to peptide signals (Fig. 1B and C). The addition of higher concentrations of sXIP merely induced CMV formation in Δ*comS,* suggesting that Δ*comS* was unable to induce the autolysis required for CMV induction in response to 10 nM of sXIP due to the defect in the positive feedback loop of the native XIP precursor expression (Fig. S1). Differences in the response of the WT and Δ*comS* to sXIP were also observed in growth, where Δ*comS* required higher concentrations of sXIP to inhibit growth (Fig. S2A and B).

Δ*lytF*, which lacks autolysin in the SigX regulon, produced significantly fewer CMVs than WT in the presence of peptide signals, although it slightly induced CMV production in response to sCSP (Fig. 1B and C). CMV production was restored in the *lytF*-complemented strain (Fig. 1B and C). In contrast to sCSP, CMV production was induced by sXIP in Δ*comDE* and Δ*cipB* strains. These results are consistent with the previous report showing that *comDE* and *cipB* are not required for the induction of *lytF* expression in CDM (10). LytF is an autolysin expressed in a subpopulation of cells that triggers cell death (7). Hence, it is most likely that CMVs were formed through cell death. We also confirmed that cell death is induced by sCSP in a LytF-dependent manner (Fig. S3). Dead cells were not detected by propidium iodide when sXIP was added (Fig. S4), likely due to disintegration of dead cells. Indeed, cell disintegration induced by sXIP was clearly observed with TEM, and this process was found to be LytF-dependent (Fig. S5).

**Fig 2:**
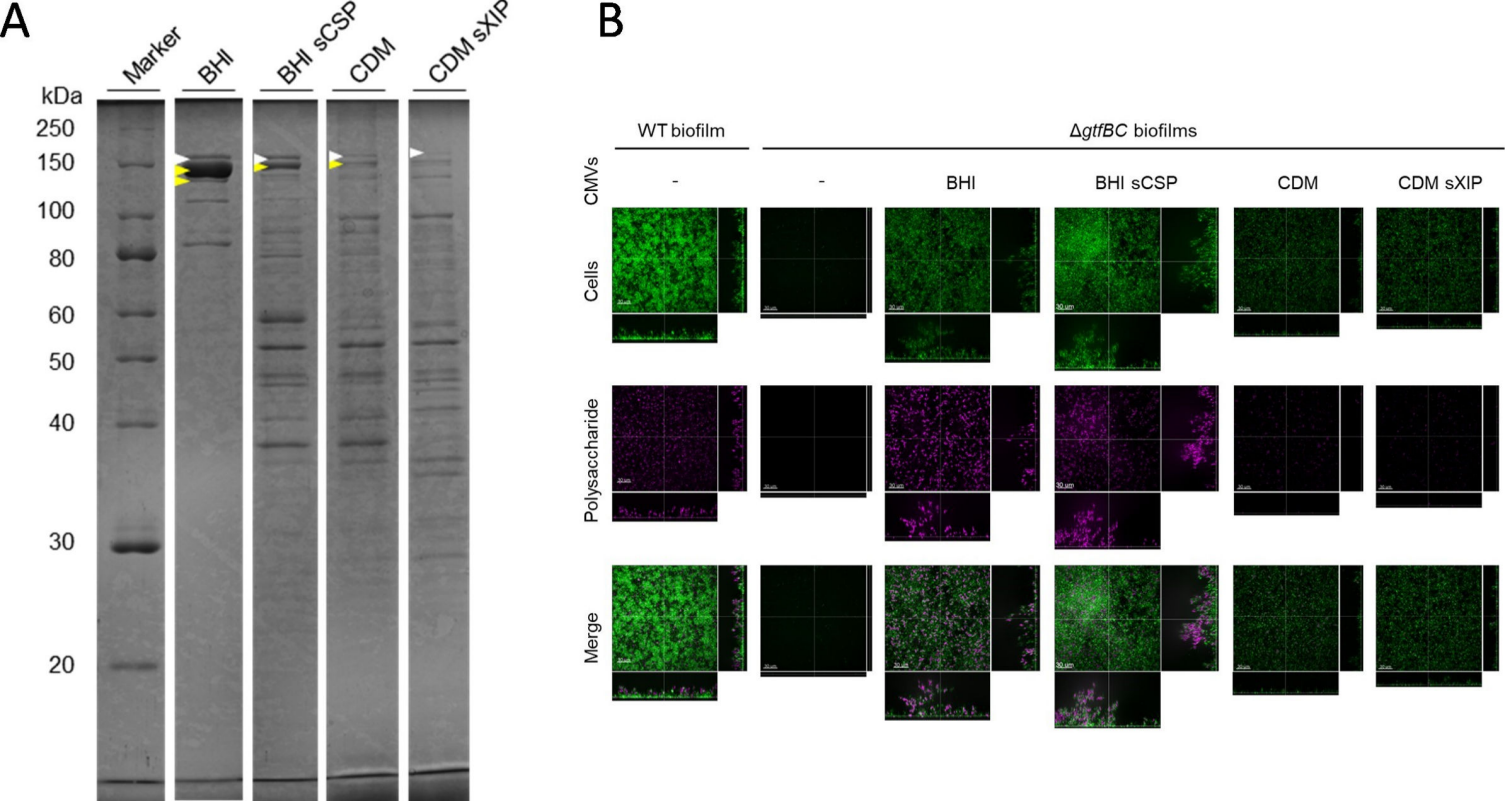
Identification of CMV proteins and functional evaluation. (**A**) CMV proteins released by *S. mutans* UA159 WT after 24 h culture were separated by SDS-PAGE. White and yellow arrows indicate Gtf-I and Gtf-SI, respectively. The main bands were identified by MALDI-TOF/MS. The uncropped image of the SDS-PAGE is shown in Fig. S6. (**B**) *S. mutans* UA159 WT and Δ*gtfBC* were cultured in BHI supplemented with 0.25% (w/v) sucrose. The CMVs derived from *S. mutans* UA159 WT were added to the medium at a concentration of 5 µg/mL of protein. The data show 2D images of the bottom of biofilms and side views. Cells (SYTO 9) and extracellular polysaccharides (Alexa fluor 594) are shown in green and magenta, respectively. Scale bars indicate 20 µm.

In general, the MV content differs depending on the culture conditions. To examine the CMVs isolated under different culture conditions, we analyzed the CMV proteins using SDS-PAGE and identified the main proteins using MALDI-TOF/MS (Fig. 2A; Fig. S6). Consistent with a previous report (15), Gtf-I and Gtf-SI were detected in the CMVs (Fig. 2A; Fig. S6). Addition of sCSP to BHI induced significant changes in the CMV protein profile (Fig. 2A). Translation elongation factors (EF-G and EF-Tu) and DNA recombinase (RecA) were detected in the CMVs produced in the BHI sCSP (Fig. S6). *recA* is a SigX regulon gene for which expression is induced in a subpopulation of cells cultured in BHI (8). Thus, the CMVs derived from *lytF*-expressing cells reflected the protein profiles of *sigX*-expressing cells. Cytoplasmic proteins involved in metabolism (CitB, CapP, Pgk, and AckA) and transcription (RpoBC) were detected in CMVs produced in CDM and CDM sXIP (Fig. S6). The extracellular functions of these proteins are unclear, but some of these proteins have also been detected in MVs from *E. coli* and *Staphylococcus aureus* (16, 17). Whether those proteins affect the function of CMVs is an interesting topic to be explored further. Additionally, in CDM sXIP, Pgk detected in the cell sample was not detected in the CMVs (Fig. S6), implying that certain proteins were enriched in CMVs.

Because CMVs from *S. mutans* promote biofilm formation (18), we evaluated the function of CMVs in biofilm induction. *S. mutans* forms insoluble polysaccharide-dependent biofilms in the presence of sucrose (19). A Δ*gtfBC* strain, which cannot form biofilms due to disruption of insoluble polysaccharide synthases, was used to examine whether CMVs can complement the function of these enzymes in biofilm formation. Our results show that CMVs produced in BHI and BHI sCSP induced biofilm formations (Fig. 2B and Fig. S7). CMVs produced in CDM and CDM sXIP had little effect on biofilm formations, compared with BHI and BHI sCSP, which may be due to the low content of Gtfs (Fig. 2A and B; Fig. S7). CMVs derived from the Δ*gtfBC* strains did not stimulate biofilm formation, except for those derived from CDM sXIP, which slightly promoted biofilm formation regardless of the presence of Gtfs (Fig. S8). In addition to extracellular polysaccharides, extracellular DNA has been reported to contribute to *S. mutans* biofilm formation (20). Since many cells in CDM sXIP were disintegrated (Fig. 1E; Fig. S5), it is possible that the CMVs were rich in extracellular DNA derived from dead cells, which promoted biofilm formation.

Cell death is a universal route of MV formation in bacteria (5). For a population to benefit from cell death, it is important that cell death is triggered only in a subpopulation of cells. We demonstrated that MV release was triggered in a subpopulation of cells via cell-to-cell communication-mediated autolysis. It is also interesting to see if MVs are involved in signaling, since cell lysis has been reported to be critical in releasing XIP precursor into the extracellular environment (21). Our findings could be conserved in other streptococci in which autolysins are regulated by the Com system (22, 23).

## SUPPLEMENTAL MATERIAL

Supplemental Material spectrum.00334-25-s0001.pdf Supplemental materials and methods, tables, and figures.

ASM does not own the copyrights to Supplemental Material that may be linked to, or accessed through, an article. The authors have granted ASM a non-exclusive, world-wide license to publish the Supplemental Material files. Please contact the corresponding author directly for reuse.
