# Supplementary material for: Membrane vesicle production via cell-to-cell communication-induced autolysis in Streptococcus mutans
Source: Microbiol Spectr. 2025 May 23;13(7):e00334-25. doi: 10.1128/spectrum.00334-25 (PMC12211075; doi:10.1128/spectrum.00334-25)
Supplement: Supplemental Material — s and methods, tables, and figures. [file spectrum.00334-25-s0001.pdf]

Membrane vesicle production via cell-to-cell communication-induced autolysis in *Streptococcus mutans*

Ryo Nagasawa[1,#,†], Tamami Ito[2,#], Chika Yamamoto[1], Mio Unoki[2], Nozomu Obana[3,4], Nobuhiko Nomura[4,5,6], Masanori Toyofuku[4,5,6*]

1. Graduate School of Life and Environmental Sciences, University of Tsukuba, 1-1-1 Tennodai, Tsukuba, Ibaraki 305-8572, Japan
2. Graduate School of Science and Technology, University of Tsukuba, 1-1-1 Tennodai, Tsukuba, Ibaraki 305-8572, Japan
3. Faculty of Medicine, Transborder Medical Research Center, University of Tsukuba, 1-1-1 Tennodai, Tsukuba, Ibaraki 305-8577, Japan
4. Microbiology Research Center for Sustainability, University of Tsukuba, 1-1-1 Tennodai, Tsukuba, Ibaraki 305-8572, Japan
5. Faculty of Life and Environmental Sciences, University of Tsukuba, 1-1-1 Tennodai, Tsukuba, Ibaraki 305-8572, Japan
6. Tsukuba Institute for Advanced Research, University of Tsukuba, 1-1-1 Tennodai, Tsukuba, Ibaraki, 305-8577, Japan

# Co-first authors
† Present address: Department of Microbiology and Immunology, Aichi Medical University School of Medicine, 1-1 Karimata Yazako, Nagakute, Aichi 480-1195, Japan
* Corresponding author

Supplementary information:

- Materials and Methods
- Supplementary Figures S1-S8
- Supplementary Tables S1 and S2
- References

Materials and Methods

Strains and culture conditions

The bacterial strains used in this study are listed in Table S1. We cultured these strains in BHI broth (Difco Laboratories, MI, USA) or CDM in an aerobic atmosphere containing 5% CO_2 at 37°C. The composition of CDM in this study was identical, referring to the paper by van de Rijn and Kessler [1] .

Construction of deletion mutant

The primers used to construct the Δ*comC* strain are listed in Table S2. Sequence information was obtained from the Kyoto Encyclopedia of Genes and Genomes (KEGG) database. The deletion mutant strain was constructed by replacing the target gene with a spectinomycin resistance gene (*aad9*) using a DNA fragment prepared by overlap-extension PCR as described below. The upstream and downstream sequences of the *comC* gene were amplified from the genomic DNA of *S. mutans* UA159. The *aad9* gene was amplified from pDL278 [2]. The amplicons were connected by overlap-extension PCR and introduced into competent cells of *S. mutans* UA159. Genetic competence was induced by adding 1 μM of sCSP to *S. mutans* UA159 cultured to early-log phase in BHI. The transformants were screened on Mitis Salivarius (MS) agar (Difco Laboratories, MI,

USA) plates with spectinomycin (200 μg/ml). Insertion of the mutation into the target region was confirmed by colony PCR and DNA sequencing.

CMVs purification and quantification

Overnight cultures were diluted to an OD_{600} of 0.01 with fresh medium and then cultured in an aerobic atmosphere containing 5% CO_2 at 37°C for 24 h. sCSP and sXIP were added at the start of culture at final concentrations of 1 μM and 10 nM, respectively. After removing the cells from the culture by centrifugation and filtration with a 0.45 μm pore size PVDF filter (Merck, Darmstadt, Germany), the samples were ultracentrifuged for 1 h at 150,000 × g, 4ºC. The pellets were resuspended in sterile phosphate-buffered saline (PBS). After further purification, the CMV pellets were resuspended in 45% iodixanol (OptiPrep™, Serumwerk Bernburg AG, Bernburg, Germany) in HEPES-NaCl buffer and subjected to density gradient ultracentrifugation with 45%−10% iodixanol. The CMVs were quantified using a plate reader (BioTek Cytation5, Agilent, CA, USA) after labelling with FM®1-43FX (Invitrogen, MA, USA). FM®1-43FX was excited at 498 nm and the fluorescence was detected at 598 nm. The results were standardized by the OD_{600} of the 24-h culture solution.

Quantification of dead cells

Overnight cultures were diluted to an OD_{600} of 0.01 with fresh medium and then cultured by a plate reader (BioTek Cytation5, Agilent, CA, USA) in an aerobic atmosphere containing 5% CO_2 at 37°C. sCSP or sXIP was added at the start of culture. Dead cells were labeled with 500 nM SYTOX® Green (Invitrogen, MA, USA) or 6 µg/ml Propidium Iodide (Invitrogen, MA, USA) and excited by 488 nm and 550 nm lasers, respectively. The fluorescence emissions were detected at 590 to 610 nm for SYTOXGreen and 587 to 623 nm for Propidium Iodide, every 30 min for 24 h.

Microscopy

Purified CMVs were negatively stained with uranyl acetate and observed under a JEM-1400Flash electron microscope (JEOL, Tokyo, Japan) at the Hanaichi Ultrastructure Research Institute. Ultrathin sections of the bacterial cells were prepared using the following procedure and then observed under a transmission electron microscope (TEM; H-7650, Hitachi, Tokyo, Japan). Cells collected by centrifugation were fixed with 2% glutaraldehyde. After initial fixation, the samples were washed three times with PBS, and further treated with 2% OsO4. The fixed samples were dehydrated by sequential treatment with 50, 70, 90, and 100% ethanol. The samples were then replaced with a propylene

oxide solution and embedded in epoxy resin (Epon 812). The samples were sectioned using an ultramicrotome, placed on carbon film-coated grids (Cu400CN, Alliance Biosystems, Japan), and negatively stained with an EM stainer (Nisshin EM, Tokyo, Japan).

CMVs-attached cells were observed by scanning electron microscopy (SEM). Cells cultured for 24 h were attached to poly-L-lysine-coated coverslips and fixed in 2% glutaraldehyde. The fixed samples were dehydrated with 50, 70, 90, and 100% ethanol, replaced with tert-butyl alcohol, and freeze-dried. The sample surface was coated with platinum-palladium by ion sputtering and observed by SEM (S4200, Hitachi High-Technologies Corporation, Tokyo, Japan).

SDS-PAGE and protein identification

The CMV solutions were mixed with an equal volume of 2×SDS-PAGE sample buffer (100 mM Tris-HCl [pH 6.8], 20% glycerol, 4% SDS, 5% 2-mercaptoethanol, and 0.2% bromophenol blue) and heated to 95°C for 5 min. Samples containing equal amount of protein (5 µg/well) were separated by 12% polyacrylamide gel electrophoresis. The protein bands were visualized by Coomassie Brilliant Blue staining. The bands were then cut from the gel and the proteins were treated with trypsin. A carbamidomethyl group was

added to the -SH group of the cysteine residues of the peptides and the peptides were then analysed by MALDI-TOF/MS. The obtained mass spectra were analysed by a MASCOT database search (Matrix Science Ltd., London, UK) and the protein with the highest score for *S. mutans* UA159 was assigned to the band.

Confocal microscopy and image quantification

Overnight cultures of *S. mutans* UA159 Δ*gtfBC* were resuspended by 100-fold dilution with fresh BHI supplemented with 0.25% (w/v) sucrose (BHIs). The cell suspensions were transferred to glass bottom dishes (Matsunami, Osaka, Japan) and CMVs were added. For biofilm assays we added CMV at a final concentration of 5 μg/ml according to Senpuku et al. [3]. To visualize extracellular polysaccharides, 1 μM of alexa fluor 594-dextran conjugate (Invitrogen, MA, USA) was added before the start of culture. Biofilms were formed in an aerobic atmosphere containing 5% CO_2 at 37°C for 12 h. After removing planktonic cells, the biofilm cells were stained with SYTO 9 for 30 min and washed twice. Biofilms were treated with Clearing Reagent iCBiofilm-H1 [for Biofilm] (Tokyo Chemical Industry Co., Ltd., Tokyo, Japan) to observe deeper areas. We acquired 3D images of biofilms using an inverted spinning disk confocal microscope (SpinSR10, Olympus, Tokyo, Japan) with an Apochromat 60×/1.3 silicone oil-immersion

objective lens. Z-stacks were acquired at 0.31 μm intervals. SYTO 9 and Alexa 594 were excited by 488 nm and 561 nm lasers, respectively, and the emissions were detected (500 to 550 nm for SYTO 9 and 665 to 705 nm for Alexa 594). For image analysis, we calculated the area where SYTO 9 and Alexa 594 fluorescence were detected using Imaris (Oxford instruments, Abingdon-on-Thames, UK).

Statistics

Statistical differences were analysed using a one-way analysis of variance, Tukey's HSD test, or Dunnett's test (SPSS Statistics, IBM, NY, USA). Three independent experiments were performed. A p value of < 0.05 was considered statistically significant.

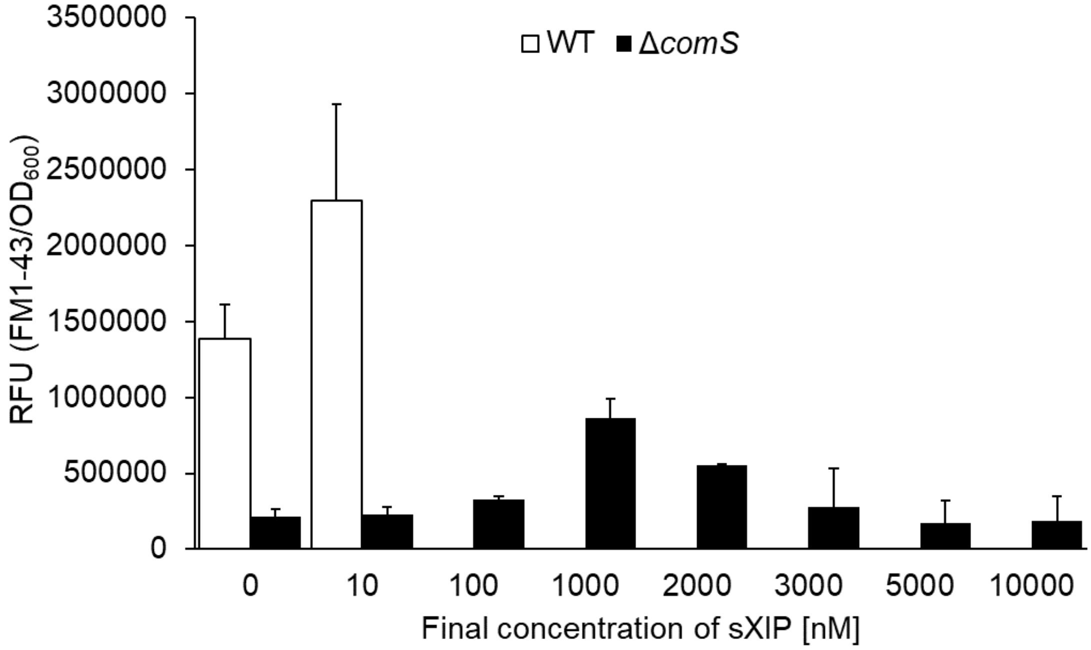

Fig. S1 Quantification of CMVs produced by Δ*comS*. Δ*comS* were cultured in CDM for 24 h. sXIP were added at the start of culture at final concentrations of 10, 100, 1000, 2000, 3000, 5000, and 10000 nM. CMVs were collected from the supernatant, stained with FM1-43FX and quantified. As a reference, CMVs produced from WT with and without 10 nM of sXIP were also quantified.

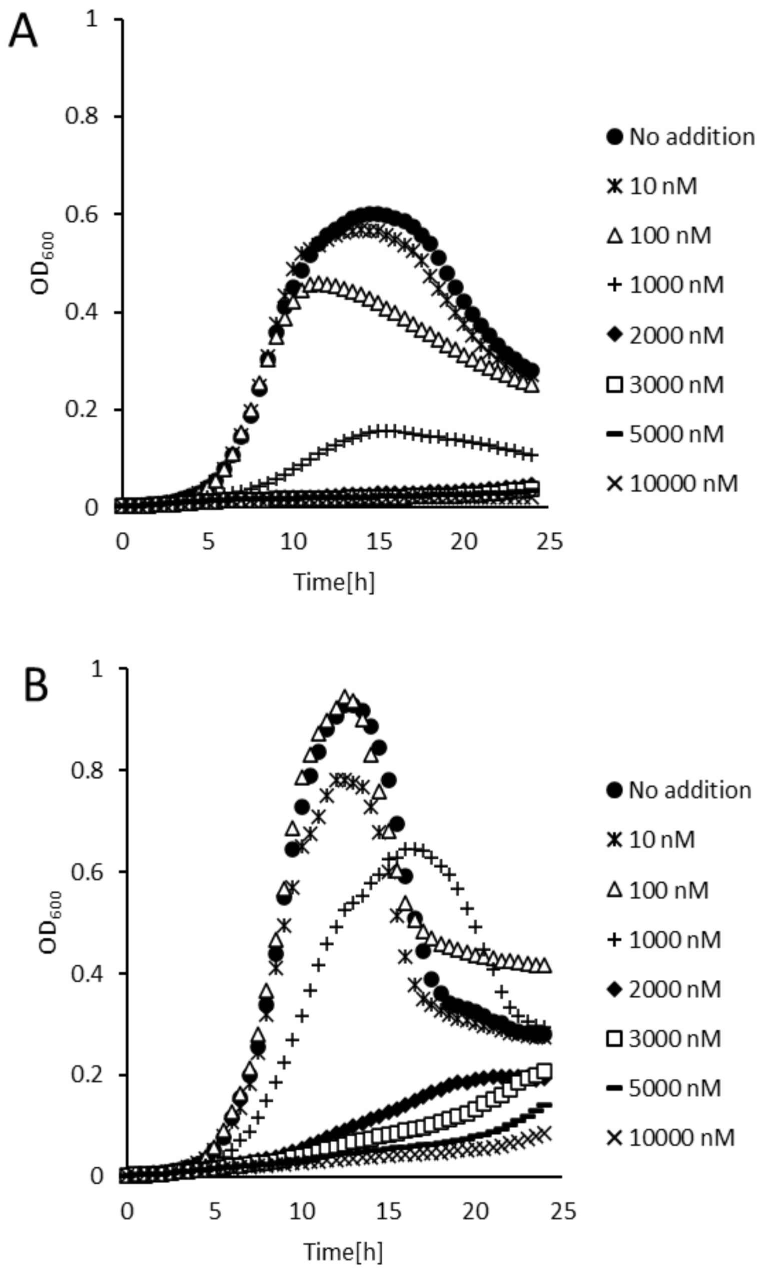

Fig. S2 Growth curves of *S. mutans* UA159 WT (A) and Δ*comS* (B). Cells were cultured in CDM and OD_{600} was measured every 30 min. sXIP were added at the start of culture at final concentrations of 10, 100, 1000, 2000, 3000, 5000, and 10000 nM.

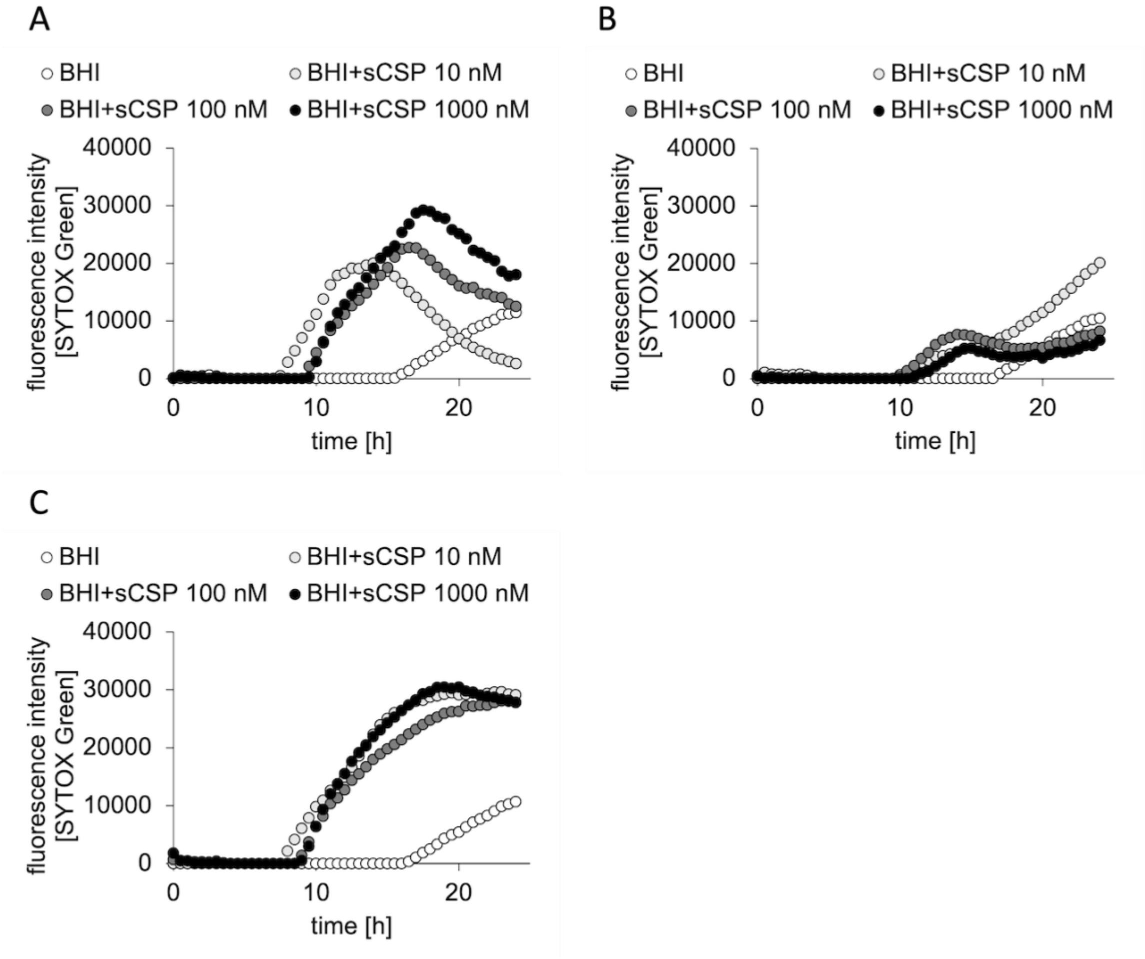

Fig. S3 Quantification of dead cells induced by sCSP. Cells were cultured in BHI. sCSP was added at the start of culture at final concentrations of 10, 100, and 1000 nM. Dead cells and extracellular DNA were stained with SYTOX Green. (A)WT, (B)Δ*lytF*, (C)*lytF* comp.

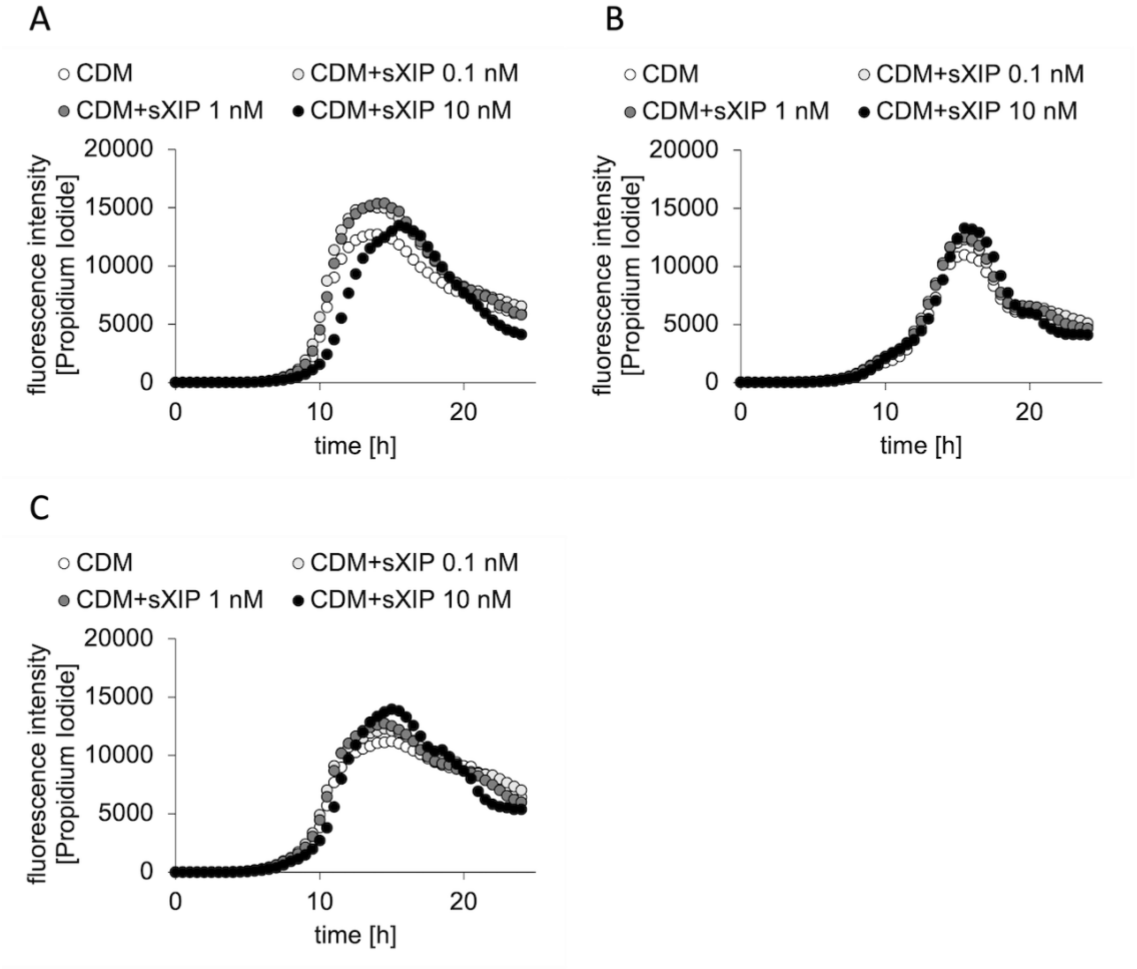

Fig. S4 Quantification of dead cells induced by sXIP. Cells were cultured in CDM. sXIP was added at the start of culture at final concentrations of 0.1, 1, and 10 nM. Dead cells and extracellular DNA were stained with Propidium iodide. (A)WT, (B)Δ*lytF*, (C)*lytF* comp.

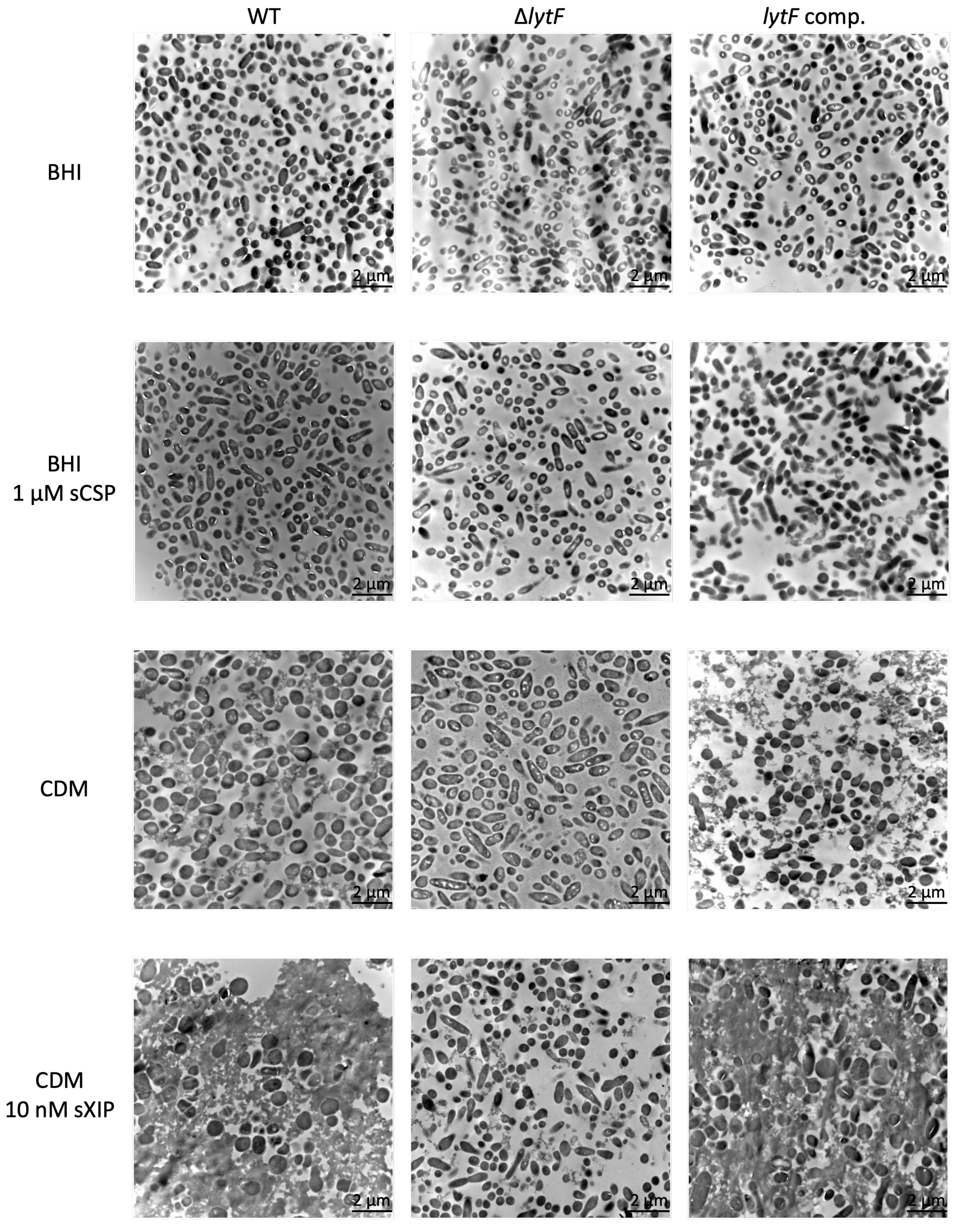

Fig. S5 TEM observation of ultrathin sections of cells. Ultrathin sections of cells cultured for 24 h were prepared and observed by TEM.

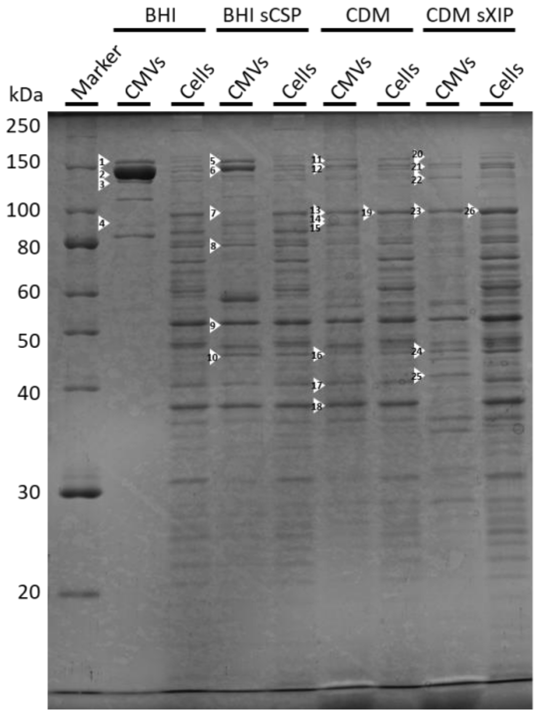

Band No.	Protein
1, 5, 11, 20	Gtf-I, Glucosyltransferase-I
2, 3, 5, 6, 12	Gtf-SI, Glucosyltransferase-SI
4, 7, 15	Ftf, Levansucrase
8	EF-G, Translation elongation factor G
9	EF-Tu, Translation elongation factor Tu
10, 16, 24	RecA, Recombination protein RecA
13, 19, 23, 26	CitB, Aconitate hydratase
14	CapP, Phosphoenolpyruvate carboxylase
17, 18	Pgk, Phosphoglycerate kinase
17, 25	AckA, Acetate kinase
21	RpoB, DNA-directed RNA polymerase subunit beta
22	RpoC, DNA-directed RNA polymerase subunit beta'

Fig. S6 SDS-PAGE and MALDI-TOF-MS of CMVs and cells. Each lane contained 5 µg of protein. Bands were cut out and proteins were identified by MALDI-TOF-MS. The bands for CMVs are also shown as Fig. 2A.

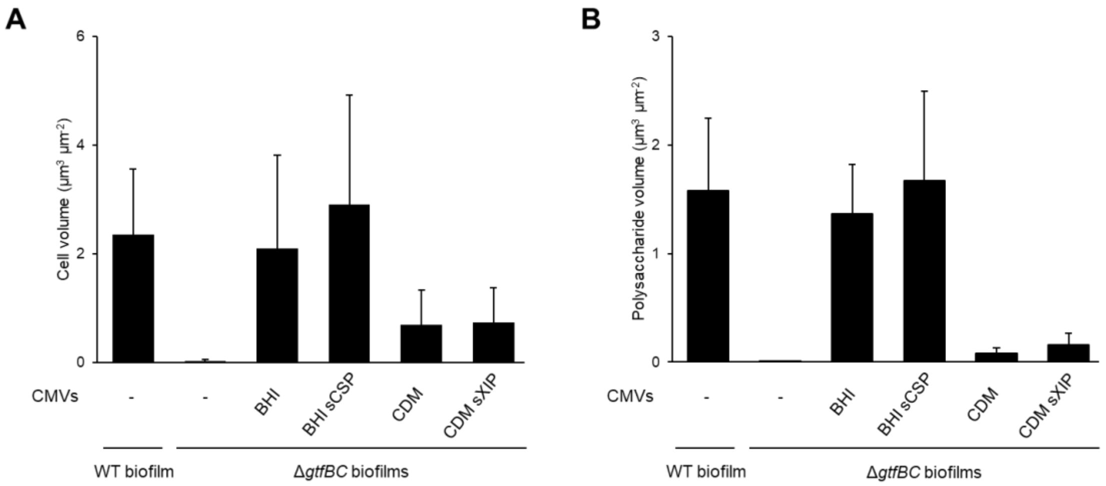

Fig. S7 Biofilm volume per a unit area. (A) The volumes occupied by cells (SYTO 9) and (B) polysaccharides (Alexa fluor 594) were calculated in Imaris from the 3D image of biofilms. These data represent the means ± SD of the quantification values from three independent experiments.

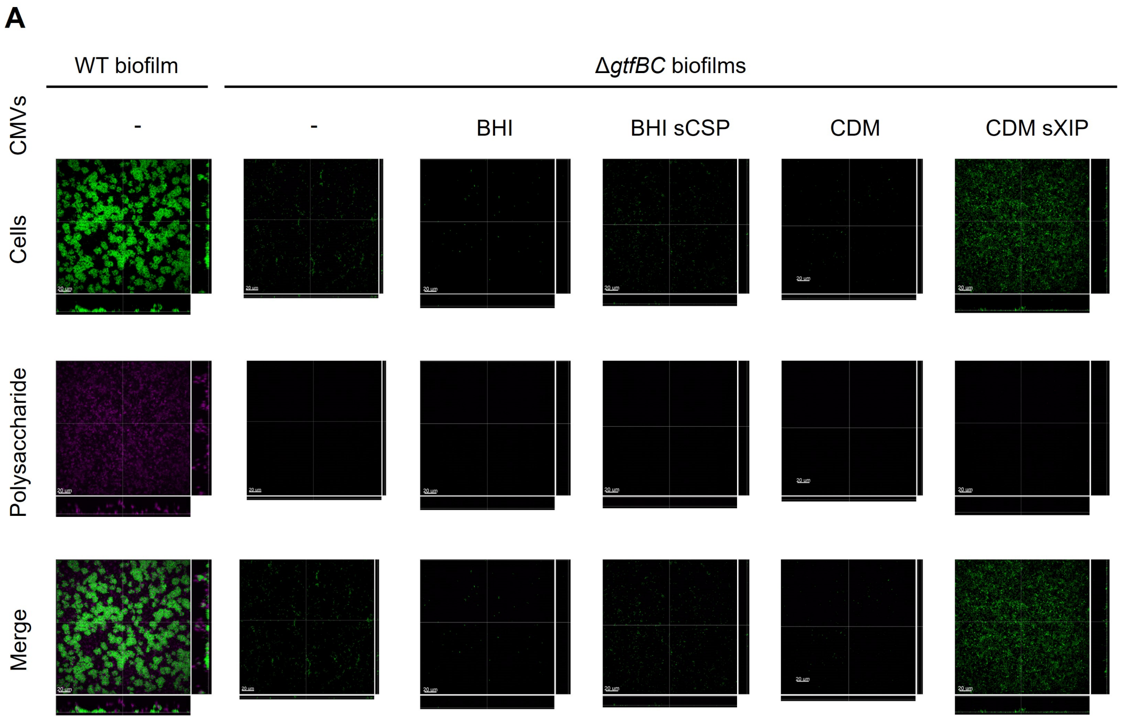

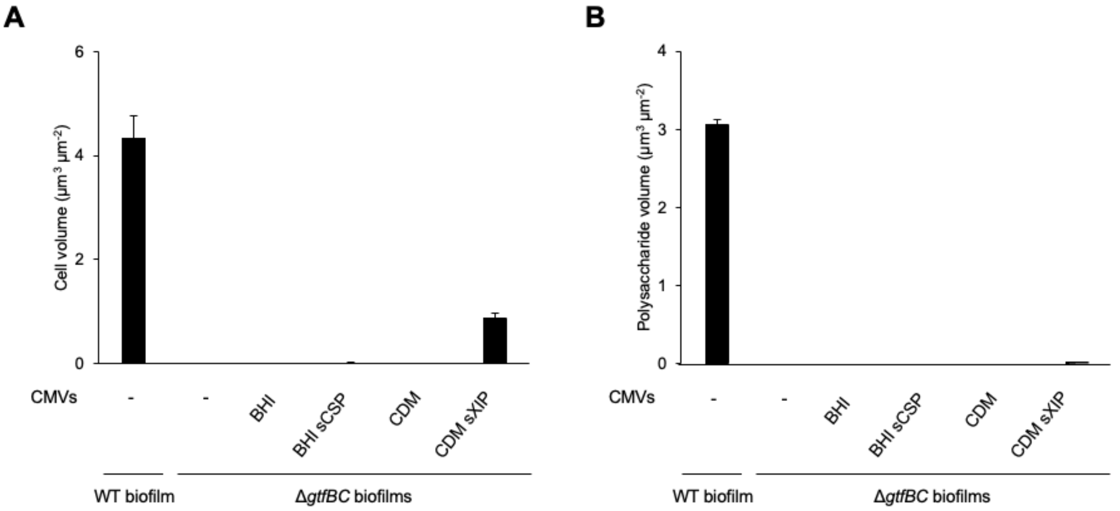

Fig. S8 Evaluation of biofilm induction by CMVs from *S. mutans* UA159 Δ*gtfBC*. *S. mutans* UA159 WT and Δ*gtfBC* were cultured in BHI supplemented with 0.25% (w/v) sucrose. The CMVs derived from *S. mutans* UA159 Δ*gtfBC* were added to the medium at concentration of 5 μg/mL of protein. (A) The data show 2D images of the bottom of biofilms and side views of the 3D image. Cells (SYTO 9) and extracellular polysaccharides (Alexa fluor 594) are shown in green and magenta, respectively. (B) The volumes occupied by cells (SYTO 9) and (C) polysaccharides (Alexa fluor 594) were calculated using Imaris from the 3D image of biofilms. These data represent the means ± SD of the quantification values from three independent experiments.

Table S1 Bacterial strains and plasmids

Strain or plasmid	Properties	Reference
Strain		
S. mutans		
UA159	Wild-type	[4]
Δ*comC*	UA159 *comC* deletion mutant; *aad9*	This study
Δ*comDE*	UA159 *comDE* deletion mutant; *ermBP*	[5]
Δ*cipB*	UA159 *cipB* deletion mutant; *ermBP*	[5]
Δ*comR*	UA159 *comR* deletion mutant; *ermBP*	[5]
Δ*comS*	UA159 *comS* deletion mutant; *ermBP*	[5]
Δ*sigX*	UA159 *sigX* deletion mutant; *ermBP*	[5]
Δ*lytF*	UA159 *lytF* deletion mutant; *ermBP*	[5]
lytF comp	UA159 *lytF* deletion mutant; *ermBP*, *SMU_437*:: P_{lytF} *lytF aph3*	[5]
Δ*gtfBC*	UA159 *gtfB* and *gtfC* disruption; *ermAM*, *aphAIII*	[3]
Plasmid		
pDL278	*aad9*	[2]

Table S2 Primers for mutant construction

Primer	Sequence
comC up Fw	GCTAAGAATTGTTTTATACTAATCCAACTTGATC
comC up Rv	CTCTTGCCAGTCACGTTACGTTATTAGGCTGGTAATGATAGTTTCAGAACATC
comC down Fw	CGATAAAATCCGATTAAGATACTGCCTACGATAGGCTAACATTGGAATAAAACAAGGCTG
comC down Rv	GGGCAATCATATTCTTTATCTTGGATG
Spec Fw	CTAATAACGTAACGTGACTGGCAAG
Spec Rv	GTAGGCAGTATCTTAATCGGATTTTATCG

References

1. Van De Rijn I, Kessler RE. Growth characteristics of group A streptococci in a new chemically defined medium. *Infect Immun* 1980 Feb;27(2):444-8.
2. LeBlanc DJ, Lee LN, Abu-Al-Jaibat A. Molecular, genetic, and functional analysis of the basic replicon of pVA380-1, a plasmid of oral streptococcal origin. *Plasmid* 1992 Sep;28(2):130-45.
3. Senpuku H, Nakamura T, Iwabuchi Y, Hirayama S, Nakao R, Ohnishi M. Effects of Complex DNA and MVs with GTF Extracted from *Streptococcus mutans* on the Oral Biofilm. *Molecules* 2019 Aug 28;24(17):3131.
4. Ajdić D, McShan WM, McLaughlin RE, Savić G, Chang J, Carson MB, Primeaux C, Tian R, Kenton S, Jia H, Lin S, Qian Y, Li S, Zhu H, Najar F, Lai H, White J, Roe BA, Ferretti JJ. Genome sequence of *Streptococcus mutans* UA159, a cariogenic dental pathogen. *Proc Natl Acad Sci U S A*. 2002 Oct 29;99(22):14434-9.
5. Nagasawa R, Yamamoto T, Utada AS, Nomura N, Obana N. Competence-Stimulating-Peptide-Dependent Localized Cell Death and Extracellular DNA Production in *Streptococcus mutans* Biofilms. *Appl Environ Microbiol* 2020 Nov 10;86(23):e02080-20.
